# CLINICAL CORRELATION OF MMSE AND TAU RESULTS FROM BLOOD OR IMAGING REPORTS

**DOI:** 10.1093/geroni/igae098.3935

**Published:** 2024-12-31

**Authors:** Surya Sunil, Malini Nair, Rui Huang, Viviana Obando, Kyra Ferreira, Jennifer Glynn, Anil Nair

**Affiliations:** Boston Neuropsychiatry, Boston, Massachusetts, United States; Boston Neuropsychiatry, Boston, Massachusetts, United States; Boston Neuropsychiatry, Boston, Massachusetts, United States; Boston Neuropsychiatry, Boston, Massachusetts, United States; Boston Neuropsychiatry, Boston, Massachusetts, United States; Boston Neuropsychiatry, Boston, Massachusetts, United States; Boston Neuropsychiatry, Boston, Massachusetts, United States

## Abstract

**Background:**

A novel blood test for Alzheimer pathology is now available. Abnormal tau measurements from blood or brain PET may indicate brain tau. We analyzed the correlation of Mini Mental Status Examination (MMSE) to tau results disclosed to patients in a memory clinic.

**Objectives:**

To assess the correlation of blood or PET tau positivity to MMSE test scores. To evaluate an MMSE score cut-off that translates optimally to tau positivity.

**Method:**

Retrospective observational chart review of patients attending a memory clinic in Boston, MA, with a MMSE test score and blood tau or tau-results were included for analysis. Baseline MMSE and a binary blood or PET scan result were analyzed with covariates age, sex and race. Spearman correlation of MMSE to blood/PET tau. Kruskal Wallis/Chi square tests for relationship of age, sex, gender and education with MMSE score and tau-PET status. ROC analyses and sensitivity/specificity were obtained.

**Results:**

We disclosed 45 positive and 10 negative tau results. 58.2% were female and 82.7% were white. Mean age was 70.12 ± 14.17, mean MMSE was 21.35±7.36. Tau abnormality trended negatively with MMSE score (r= -0.26, p=0.07). A cut point of 28 for MMSE estimated tau deposition in the brain by PET scan or blood tau, with an AUC of 0.694 (95%CI 0.48 to 0.90).

**Conclusion:**

In a small pilot study, tau blood or PET results trended negatively with MMSE scores, possibly indicating a ceiling effect as we only analyzed very mildly impaired subjects.

